# FEASIBILITY AND MECHANISM ENGAGEMENT OF A MINDFULNESS MOBILE APP FOR CAREGIVERS: FINDINGS FROM AN NIH STAGE 1B TRIAL

**DOI:** 10.1093/geroni/igae098.0339

**Published:** 2024-12-31

**Authors:** Evan Plys, Emily Woodworth, Morgan Seward, Elizabeth Allen, Isabella Carroll, Jen Huberty, Ana-Maria Vranceanu

**Affiliations:** Massachusetts General Hospital, Boston, Massachusetts, United States; Massachusetts General Hospital, Boston, Massachusetts, United States; Massachusetts General Hospital, Boston, Massachusetts, United States; Massachusetts General Hospital, Boston, Massachusetts, United States; Massachusetts General Hospital, Boston, Massachusetts, United States; Fit Minded Inc., Phoenix, Arizona, United States; Massachusetts General Hospital, Boston, Massachusetts, United States

## Abstract

A large body of research has tested psychological interventions with dementia caregivers. Yet, this literature still lacks clear understanding of mechanisms of action for psychological outcomes. This NIH Stage 1B study tested the feasibility and mechanism engagement of a theory-based mindfulness mobile-app (Healthy Minds Program [HMP]) for stress, depression, and anxiety among caregivers; mindfulness skill was the proposed mechanism for each outcome. Stressed caregivers were randomized to HMP (n=40) or a podcast control (n=40). Groups did not vary by age, gender, race, or caregiver type. Among all participants (N=80), benchmarks were met for recruitment (90% eligible caregivers enrolled), data collection (98% with no questionnaires fully missing), and attrition (95% completed post-tests). For caregivers completing HMP, benchmarks were met for treatment satisfaction and credibility, but not for expectancy or adherence (63% completed 52.5 minutes weekly). Using Wilcoxon signed-rank/paired-samples t-tests, caregivers receiving HMP evidenced significant within-group improvements in mindfulness (Applied Mindfulness Process Scale; Cohen’s d=1.16). Significant within-group differences were observed for stress (Perceived Stress Scale; Cohen’s d=.91), depression (Hospital Anxiety and Depression Scale [HADS]; Cliff’s delta=.34), and anxiety (HADS; Cohen’s d=.78). Change in mindfulness significantly related with change in stress (r=.38), depression (r=.42), anxiety (r=.33). Findings suggest the HMP mobile-app was feasible and led to moderate to large within-group effects for mindfulness and clinical outcomes among caregivers. Moderate correlations between change scores confirmed the conceptual model with mindfulness as a proposed mechanism of action. We discuss protocol refinements (e.g., changes to dose for mobile-app) and preparation for a Stage 2 efficacy trial.

